# 908 Social Vulnerability Index and Burn Injury - A Better Tool to Target At-Risk Patients?

**DOI:** 10.1093/jbcr/iraf019.439

**Published:** 2025-04-01

**Authors:** Meredith Hanrahan, Jeffrey Anderson, Daniel Wiese, Morgan Palumbo, Kevin Henry

**Affiliations:** Temple University Hospital; Temple University Hospital; Temple University; Lewis Katz School of Medicine; Temple University

## Abstract

**Introduction:**

Social vulnerability is a community’s predisposition to delayed or inadequate recovery in response to various situations including environmental, health or economic disasters. The Center for Disease Control (CDC) uses the social vulnerability index (SVI) to identify communities experiencing social vulnerability and therefore those at higher risk for health inequalities. The purpose of this study was to determine if SVI on a census tract level can be used as a predictor of hospital admission for burn treatment and can further be used to predict outcomes in burn patients.

**Methods:**

In this retrospective cohort study, medical records were reviewed for 167 patients admitted to the burn unit at an ABA-verified urban academic burn center between 2020 to 2023. Inclusion criteria were burn injury of any total body surface area and age ≥ 18. We stratified subjects based on five SVI measures using patient home address and tract level SVI: overall SVI, socioeconomic status, household characteristics, racial & ethnic minority status, and housing type & transportation. Each census tract receives a ranking for each theme as well as the overall ranking that is calculated by adding up individual themes and converting the summated score into a percentile rank, ranging from 0 to 1, with higher values indicating greater vulnerability. Subjects were divided into SVI quartiles (0 to 0.25, 0.25 to 0.50, 0.50 to 0.75, 0.75 to 1.0), with 1st quartile (0 to 0.25) being the least vulnerable and 4th quartile (0.75 to 1.00) being the most vulnerable. Burn cases and SVI quartile were compared by Pearson’s Chi-Squared Test.

**Results:**

Our cohort consisted of 149 subjects. The median age was 51 years (21-90 years) and 58.3% (87) subjects were male. Of the 149 patients admitted to the burn unit, 18 (12.1%) were in the 1st quartile, 20 (13.4%) were in the 2nd quartile, 35 (23.5%) were in the 3rd quartile, and 76 (51.0%) were in the 4th quartile (p< 0.001) based on overall vulnerability. For the individual themes of socioeconomic status, household characteristics, racial & ethnic minority status, and housing type & transportation, 82 (55.0%), 75 (50.3%), 91 (61.1%), and 28 patients (18.8%), respectively, fell into the 4th quartile (p< 0.001).

**Conclusions:**

In this single-center cohort, patients admitted to the burn unit for treatment of injuries were more likely to have a higher overall SVI rating than a low SVI rating. Additionally, the majority of patients admitted to the burn unit were in the most vulnerable group for socioeconomic status, household characteristics, and racial & ethnic minority status.

**Applicability of Research to Practice:**

These findings highlight the importance of considering social vulnerability in burn care protocols and the need for targeted interventions to support at-risk populations.

**Funding for the Study:**

N/A

